# Editorial: Non-conventional Yeast in the Wine Industry

**DOI:** 10.3389/fmicb.2016.01494

**Published:** 2016-09-27

**Authors:** Albert Mas, José M. Guillamón, Gemma Beltran

**Affiliations:** ^1^Oenological Biotechnology Research Group, Department Biochemistry and Biotechnology, Faculty of Oenology, University Rovira i VirgiliTarragona, Spain; ^2^Instituto de Agroquímica y Tecnología de los AlimentosValencia, Spain

**Keywords:** non-*saccharomyces*, torulaspora, hanseniaspora, starmerella

The alcoholic fermentation of grape musts to wines is a rather complex process that involves the sequential development of microorganisms, mainly yeasts, but also filamentous fungi, lactic acid bacteria, etc. In the early stages of wine fermentation, several yeast species may be present but, as the alcohol concentration increases, *Saccharomyces* species progressively take over. The winemaking process cannot be understood without knowing how the different microorganisms leave their microbial footprint. The footprint depends on how long these microorganisms are present and their dominance during the winemaking process.

The first source of this microbial population diversity is grape, which is an ecological niche for freely proliferating microorganisms. The grapes have populations of native or indigenous yeasts that are between 10^4^ and 10^6^ cells/g of grapes, which are mainly Non-*Saccharomyces* yeasts. The populations of *Saccharomyces* are very low in grapes, although they are not completely absent. The Non-*Saccharomyces* strains have been regarded for many years as the responsible for wine spoilage and different preventive actions have been taken to avoid them. These populations change slightly when they enter in contact with the cellar environment (presses, pumps, tanks) where they join the resident microbiota. This microbiota is rare in new wineries, particularly if the equipment has not been used previously. The cellar is a good niche for *S. cerevisiae*, which becomes the main cellar-resident yeast (Beltran et al., 2002).

According with the distribution of yeasts in the grape surface, yeasts with low fermentation activity, such as *Candida* spp., *Hanseniaspora* spp., *Pichia* spp., *Rhodotorula* spp., *Kluyveromyces* spp. and *Schizosaccharomyces* spp. are predominant in grape musts and during the early stages of fermentation. *Saccharomyces cerevisiae* develops shortly afterwards, to become the dominating microorganism and completing the wine fermentation. *S. cerevisiae* strains have unique physiological properties that are not found in other yeasts. The most important is the high ability to ferment sugars vigorously to produce alcohol under both aerobic and anaerobic conditions. This aptitude allows them to colonize quickly substrates with high sugar concentrations and overgrow other yeasts (Fleet and Heard, 1993).

The role of *S. cerevisiae* yeasts is not only related with conducting the alcoholic fermentation, but is also heavily related to wine quality. The activities of the different yeast species and strains have an impact on the sensory profiles of wine by increasing its complexity and organoleptic richness (Fleet, 2003). Currently, winemakers use available commercial starters of *S. cerevisiae* to have a reproducible and predictable wine by controlling the fermentation. The selection of these commercially available starters has been based on different criteria, and many different presentations can be found. There are *S. cerevisiae* strains selected to increase aromatic expression, to ferment musts with high sugar concentration (osmophilic yeast), to resist high or low fermentation temperatures, or to survive in wines with high ethanol content, also able to perform second fermentation (for instance for sparkling wine production), among other properties. Nonetheless, the main characteristic of all of them is that they are good fermenters and they are able to finish the alcoholic fermentation. However, the use of these commercial starter cultures produces the quick take over of the fermentation and the elimination of native microorganisms and their impact on the final wines. As consequence, the massive use of these selected yeasts derives in very uniform wines with small differences. These differences are due to similar analytical values and organoleptic profiles, and thus, limiting the personality (in terms of variability and complexity) that define the typicality of a wine. Wine typicality could be defined as the characteristics that allow the identification of a wine with the territory (terroir, AOC, for instance) where it has been produced. The defense of this typicality can be done by the use of native or indigenous yeasts, as certain microbial diversity is associated to a given area (Bokulich et al., 2013). This diversity has been analyzed in different agronomic conduction systems (Setati et al.).

Nonetheless, a way of balancing control and yeast population diversity during wine fermentation is the selection of Non-*Saccharomyces* yeasts with optimal oenological traits. Non-*Saccharomyces* species contribute increasing the concentration of volatile compounds and the chemical composition of wines due to higher production of secondary metabolites that contribute to the organoleptic properties of the wines (glycerol, aromas such esters, acetates,…). Some extracellular enzymes (esterases, pectinolytic, beta-glucosidase, etc.) produced by these yeasts may be responsible for the appearance in the wine of unique properties that allow its identification from the region where is produced (reviewed in Jolly et al., 2014). The use of Non-*Saccharomyces* yeast can reduce the ethanol content as reviewed in the present issue by Ciani et al. The ethanol reduction is a critical aspect in winemaking due to climate change, which produces an increased concentration of sugars. However, the common opinion of winemakers on Non-*Saccharomyces* yeasts is that they are mostly spoilage microorganisms and thus they restrict its use. However, there is an increasing interest on Non-*Saccharomyces* yeast species for the selection of starters and their use in mixed fermentations with *S. cerevisiae*, which is probably changing the traditional bias of winemakers now.

The selection of Non-*Saccharomyces* yeast has focused on their direct positive effects on wine quality either by providing new aromas such as volatile fatty acids, esters, aldehydes, etc or by removing detrimental compounds that would affect wine quality. A couple of articles have approached this aspect in this special topic (Belda et al.; Padilla et al.). *Torulaspora delbrueckii* has been proposed to reduce volatile acidity produced by *Saccharomyces*. In this special topic, several articles are dealing with the use of this species in wine making (Ramírez et al.; Velázquez et al.; Renault et al.). Currently, there are various commercial preparations of this yeast. Another Non-*Saccharomyces* yeast that is commercially available is *Metschnikowia pulcherrima*, recommended for the release of some volatile thiols and terpenes in white wines, thus increasing their aromatic intensity. Finally, *Lachancea thermotolerans* is also commercially available to increase glycerol and lactic acid (Gobbi et al., 2013). Although there are still few commercial preparations of Non-*Saccharomyces* yeasts, they will probably increase in the near future. These include *Starmerella bacillaris*, with production of large amounts of glycerol, and its fructophilic character, which favors the end of fermentation. However, in this topic, antimicrobial activity has been described by several strains of this species (Fernandes Lemos et al.). Some species from the *Hanseniaspora* genus are also considered for future applications. In fact, in this topic a couple of articles on *H. uvarum* (Albertin et al.; Masneuf-Pomerade et al.) and another on *H. vineae* (Lleixà et al.) have been proposed as increasing the wine quality. However, some attention should be paid to some non-conventional *Saccharomyces*, which could also be relevant in terms of new activities of interest in winemaking (Pérez-Torrado et al.). Finally, the interaction between *Saccharomyces* and Non-*Saccharomyces* yeast during winemaking should be clearly known and controlled when mixed inocula are going to be used (Wang et al.).

## Author contributions

All authors listed, have made substantial, direct and intellectual contribution to the work, and approved it for publication.

### Conflict of interest statement

The authors declare that the research was conducted in the absence of any commercial or financial relationships that could be construed as a potential conflict of interest.

## References

[B1] Beltran G. Torija M. J. Novo M. Ferrer N. Poblet M. Guillamón J. M.. (2002). Analysis of yeast populations during alcoholic fermentation: a six year follow-up study. Syst. Appl. Microbiol. 25, 287–293. 10.1078/0723-2020-00097 12353885

[B2] Bokulich N. A. Thorngated J. H. Richardsone P. M. Mills D. A. (2013). Microbial biogeography of wine grapes is conditioned by cultivar, vintage, and climate. Proc. Natl. Acad. Sci. U.S.A. 111, E139–E148. 10.1073/pnas.1317377110 24277822 PMC3890796

[B3] Fleet G. H. (2003). Yeast interactions and wine flavour. Int. J. Food Microbiol. 86, 11–22. 10.1016/S0168-1605(03)00245-9 12892919

[B4] Fleet G. H. Heard G. M. (1993). Yeasts: growth during fermentation, in Wine Microbiology and Biotechnology, ed Fleet G. H.(Chur: Harwood Academic Publishers). 27–54.

[B5] Gobbi M. Comitini F. Domizio P. Romani C. Lencioni L. Mannazzu I.. (2013). *Lachancea thermotolerans* and *Saccharomyces cerevisiae* in simultaneous and sequential co-fermentation: a strategy to enhance acidity and improve the overall quality of wine. Food Microbiol. 33, 271–281. 10.1016/j.fm.2012.10.004 23200661

[B6] Jolly N. P. Varela C. Pretorius I. S. (2014). Not your ordinary yeast: non-*Saccharomyces* yeasts in wine production uncovered. FEMS Yeast Res. 14, 215–237. 10.1111/1567-1364.12111 24164726

